# Artificial Induction of Sox21 Regulates Sensory Cell Formation in the Embryonic Chicken Inner Ear

**DOI:** 10.1371/journal.pone.0046387

**Published:** 2012-10-10

**Authors:** Stephen D. Freeman, Nicolas Daudet

**Affiliations:** UCL Ear Institute, University College London, London, United Kingdom; Universitat Pompeu Fabra, Spain

## Abstract

During embryonic development, hair cells and support cells in the sensory epithelia of the inner ear derive from progenitors that express Sox2, a member of the SoxB1 family of transcription factors. Sox2 is essential for sensory specification, but high levels of Sox2 expression appear to inhibit hair cell differentiation, suggesting that factors regulating Sox2 activity could be critical for both processes. Antagonistic interactions between SoxB1 and SoxB2 factors are known to regulate cell differentiation in neural tissue, which led us to investigate the potential roles of the SoxB2 member Sox21 during chicken inner ear development. Sox21 is normally expressed by sensory progenitors within vestibular and auditory regions of the early embryonic chicken inner ear. At later stages, Sox21 is differentially expressed in the vestibular and auditory organs. Sox21 is restricted to the support cell layer of the auditory epithelium, while it is enriched in the hair cell layer of the vestibular organs. To test Sox21 function, we used two temporally distinct gain-of-function approaches. Sustained over-expression of Sox21 from early developmental stages prevented prosensory specification, and abolished the formation of both hair cells and support cells. However, later induction of Sox21 expression at the time of hair cell formation in organotypic cultures of vestibular epithelia inhibited endogenous Sox2 expression and Notch activity, and biased progenitor cells towards a hair cell fate. Interestingly, Sox21 did not promote hair cell differentiation in the immature auditory epithelium, which fits with the expression of endogenous Sox21 within mature support cells in this tissue. These results suggest that interactions among endogenous SoxB family transcription factors may regulate sensory cell formation in the inner ear, but in a context-dependent manner.

## Introduction

The vertebrate inner ear comprises a series of interconnected fluid-filled cavities lined with distinct sensory patches responsible for hearing in the cochlea, and the perception of acceleration and gravity in the vestibular system. Each sensory patch contains a regular mosaic of mechanosensory hair cells, interspaced by non-sensory support cells. The entire inner ear is derived from a thickening of the head ectoderm called the otic placode. In birds and mammals, the placode invaginates to form the otic cup, which in turn closes to create a hollow vesicle known as the otocyst. The otocyst then transforms into the inner ear with its distinct sensory epithelia and their associated non-sensory compartments. The development of these different structures and their specialized cell types involves complex interplays between intercellular signalling pathways and cell-intrinsic regulators of gene expression, which are still poorly understood [1]–[4]. One such interaction appears to link two major players during inner ear development: the Notch pathway and the Sox2 transcription factor.

Notch signalling plays distinct roles during inner ear development. An early phase of Notch activity dependent on the Notch ligand Jagged1 (Jag1) promotes the formation of the prosensory domains – from which sensory epithelia develop. Subsequently, lateral inhibition mediated by the ligand Delta1-like 1 (Dll1) regulates hair cell versus support cell fate decisions within sensory epithelia – with Notch activity opposing hair cell differentiation [5], [6]. Sox2, a member of the SoxB1 subgroup of Sox (SRY related HMG box) transcription factors, is expressed in sensory progenitors and later on in support cells [7]–[9], and is required for the development of all inner ear sensory epithelia in mice [10]. Over-expression studies have shown that Sox2 can induce prosensory fate and ectopic formation of hair cells if it is transiently expressed at early stages of inner ear development [11]. However, hair cells downregulate Sox2 expression when they differentiate [11] and sustained over-expression of Sox2 prevents hair cell formation in the mammalian cochlea [12]. The parallel with the dual effects of Notch activity on hair cell formation is striking, and several studies have implicated Notch signalling in the regulation of Sox2 expression. At prosensory stages, loss of Notch activity or Jagged1 function leads to a down-regulation of Sox2 expression in prosensory domains [12]–[14]. Conversely, forced activation of the Notch pathway promotes prosensory character and Sox2 expression in the embryonic inner ear [11], [12], [15]–[17]. This suggests that the prosensory function of Notch activity could be dependent – at least in part - on its ability to maintain adequate levels of Sox2 within progenitor cells. However, additional factors are likely to impact on Sox2 function during inner ear development. Insights from neurogenesis led us to hypothesize that Sox21 could be among such factors.

During vertebrate neurogenesis, Sox2 and other members of the SoxB1 family (Sox1 and Sox3) suppress neural differentiation and contribute, along with Notch activity, to the maintenance of a pool of cycling progenitors [18]–[21]. On the other hand Sox21, a member of the closely related subgroup of SoxB2 genes (which also comprises Sox14), counteracts the effects of SoxB1 factors and promotes neural differentiation. The SoxB2 and SoxB1 proteins share a very similar DNA-binding domain, however the SoxB1 are transcriptional activators, while the SoxB2 proteins are repressors [22], [23]. Therefore, it has been proposed that the balance of SoxB1 and Sox21 expression could determine whether progenitor cells commit to neural differentiation or not [20], [22].

Previous studies have reported that *Sox21* is expressed in the embryonic chicken and mouse inner ear [23], [24], which makes it a good candidate regulator of Sox2 activity in this tissue. It has recently been reported that absence of *Sox21* in a knock-out mouse model causes mild patterning defects in the organ of Corti, but the precise role of Sox21 in this context remains unclear [24]. Here we investigated the function of Sox21 during the development of the chicken inner ear. We show that *Sox21* is expressed in sensory domains at the time of hair cell formation and that differences in the localization of *Sox21* transcripts exist between the auditory and vestibular sensory patches. Sustained over-expression of Sox21 from an early stage of ear development leads to a loss of Sox2 expression and inhibits prosensory specification. However induction of Sox21 after prosensory specification down-regulates Sox2 expression and Notch activity, strongly promoting hair cell differentiation in vestibular patches. Surprisingly, Sox21 over-expression does not have the same effect in the auditory epithelium, despite its inhibitory effect on Sox2 expression. This study identifies Sox21 as a regulator of hair cell differentiation and highlights temporal as well as regional differences in the function of SoxB transcription factors in the inner ear.

## Materials and Methods

### Animals

Fertilized White Leghorn chicken (Gallus gallus) eggs were obtained from Henry Stewart UK and incubated at 38°C and 30–80% humidity for designated times. Embryonic stages are either from Hamburger-Hamilton (HH) tables [25] or embryonic days (E), E1 corresponding to 24 hours of incubation. Embryos older than E5 were killed by decapitation. The UK Home Office and the University College London animal ethics committee approved all procedures that were performed.

### Plasmids

The following plasmid DNA constructs were used: 1) RCAS(B)-Sox21, which consists of a Myc-tagged version of the chicken Sox21 coding sequence in the replication-competent avian specific retroviral vector RCAS(B) [22]; 2) pT2K-TRE-B1-eGFP (herein named pTRE-eGFP), which consists of a cassette of bidirectional transcriptional units (one controlling transcription of eGFP, the other empty) under the control of a tetracycline-responsive element (TRE) between the left and right ends of *Tol2* [26]; 3) pT2K-TRE-B1-FP635 (herein named pTRE-FP635), which is a modified version of pTRE-eGFP where the far-red fluorescent protein FP635 (Evrogen) has been cloned in place of eGFP; 4) pT2K-TRE-B1-eGFP-Sox21 (herein named pTRE-eGFP-Sox21), which is a modified version of pTRE-eGFP in which the Myc-tagged chicken Sox21 cds was cut from RCAS(B)-Sox21-Myc with Xba1 and Sma1 and directionally cloned downstream of the empty transcriptional unit into the Nhe1/EcoRV sites of the vector multiple cloning site; 5) pCAGGS-T2TP, which consists of a transposase controlled by a CAGGS promoter and facilitated genomic integration of the *Tol2* flanked sequences [26]; 6) pT2K-TRE-B1-FP635-Sox21 (herein named pTRE-FP635-Sox21), which is the FP635 version of pTRE-eGFP-Sox21; 7) pT2K-CAGGS-rtTA-M2, which consists of the tetracycline-on activator between the left and right ends of *Tol2* [26]; 8) pBS-SK+Sox21, which was used to generate the *in situ* hybridization probe and consists of a 718 bp fragment from the Sox21 cds (position 1208–1925) [23]; 9) pT2K-Hes5::nd2eGFP, which is a promoter-less Tol2 vector in which a 0.8 kb fragment of the promoter of the mouse Hes5 gene [27] regulates the expression of a nuclear-localized and destabilized version of eGFP; 10) pT2K-Atoh1::nTomato is an Atoh1 reporter that was generated by digesting pT2K-CAGGS-nTomato with Sal1-BamH1 to remove the CAGGS promoter and replacing it with a Sal1-BamH1 digested fragment of the 3′ Math1 enhancer originally contained in the J2X-nGFP plasmid (kind gift from Dr. J. Johnson). Details of cloning procedures are available upon request.

### *In-ovo* Electroporation

Microelectroporation of the otic cup of E2 embryos was performed using a BTX ECM 830 Electro Square Porator™ as previously described [15]. The plasmid DNA constructs were purified using PureYield™ Plasmid Midiprep System kit (Promega) and used for electroporation at a final concentration ranging between 0.5 and 1 mg/ml. Further details of the experimental procedures for *in ovo* electroporation of the chicken otic cup and use of the Tol2 transposon vectors in this tissue are available in [28].

### Immunocytochemistry and *in situ* Hybridisation

The following antibodies were used: monoclonal mouse IgG1 anti-HCA (Hair Cell Antigen; supernatant used at 1∶1000) [29]; monoclonal mouse IgG2a anti-otoferlin (HCS1; used at 1∶200) [30]; rabbit anti-Serrate1 (used at 1∶100) [31] and rabbit anti-Delta1 (used at 1∶100) [32]; rabbit anti-Prox1 (used at 1∶250; Abcam AB11941); monoclonal mouse IgG1 anti-Myc (used at 1∶100; Santa Cruz 9E10); mouse monoclonal anti-beta tubulin class III (used at 1∶1000; Sigma T8578). Goat anti-mouse IgG or anti-rabbit IgG secondary antibodies conjugated to Alexa-405, 488, 546, 633, 647 (Invitrogen) were used at 1∶1000. Immunocytochemistry experiments and *in situ* hybridization for chicken Sox21 were performed as described in [15]. Specimens were analysed on a Zeiss LSM510 inverted confocal microscope.

### Cryosections

Wholemount *in situ* hybridisation samples were cryoprotected in PBS with 20% sucrose then washed in a 1∶1 solution of 20% sucrose and TissueTek™, before being embedded in TissueTek™ and frozen in liquid nitrogen. Frozen sections (20 µm) were collected using a Leica CM1850 Cryostat, mounted on SuperFrost Plus™ slides (Microm) and images were taken using a Zeiss Axioplan microscope fitted with a digital camera.

### Measurements of Sensory Patch Size

Sensory patch size was measured according to patch span, which was defined as the distance between the two furthest points within a patch. Patch span was measured using LSM Image Browser software (Zeiss) in whole-mount preparations immunostained with the prosensory marker Prox1.

### Quantification of Sox2 Expression, Hes5::nd2eGFP, and Atoh1::nTomato Fluorescence

For comparison of Sox2 expression levels in cells transfected with either pTRE-Sox21-eGFP or pTRE-eGFP, five samples of each condition were treated for 12 hours with doxycycline in vitro and processed for immunostaining with Sox2 and HCA/otoferlin antibodies. Measurement of mean intensity values for the eGFP and Sox2 channels (12-bits) were made on randomly selected support cell nuclei using ImageJ. Transfected nuclei were defined by a mean value of eGFP>mean eGFP_background_+10×stdev (typically between 150–250 raw fluorescence values, depending on the samples) and Sox2 expression levels were standardized for each sample using Z-scoring [z = (x-mean)/stdev] then pooled across each experimental condition (i.e. Sox21-GFP or GFP alone). Statistical analyses of Z-score values of Sox2 expression within cells transfected with either Sox21-GFP or GFP alone were computed using SPSS 19 for Mac. Some data did not follow a normal distribution, so nonparametric tests were used; all *p* values are two-tailed. A similar approach was used to analyse levels of nuclear Hes5::nd2eGFP fluorescence in samples transfected with pT2K-Hes5::nd2eGFP and either pTRE-Sox21-FP635 or pTRE-FP635.

For quantification of Atoh1::nTomato fluorescence, embryos electroporated with pT2K-Atoh1::nTomato and pTRE-Sox21-eGFP were treated *in ovo* at E6 with 10 µg of Dox and incubated for a further 15 or 24 hours. After fixation, inner ear tissue was dissected and processed for whole-mount imaging on a confocal microscope. Confocal stacks were collected and ImageJ was used to measure the mean red and green fluorescence values (12-bits) within the nuclei of randomly selected Atoh1-positive cells located in the basilar papilla and vestibular regions. Cells with a mean value of eGFP>mean eGFP_background_+10×stdev were categorized as Sox21-induced, and Z-score values of Atoh1::nTomato fluorescence were compared across categories of cells.

## Results

### *Sox21* Expression Coincides with Hair Cell Formation and Differs between Auditory and Vestibular Sensory Patches

**Figure 1:**
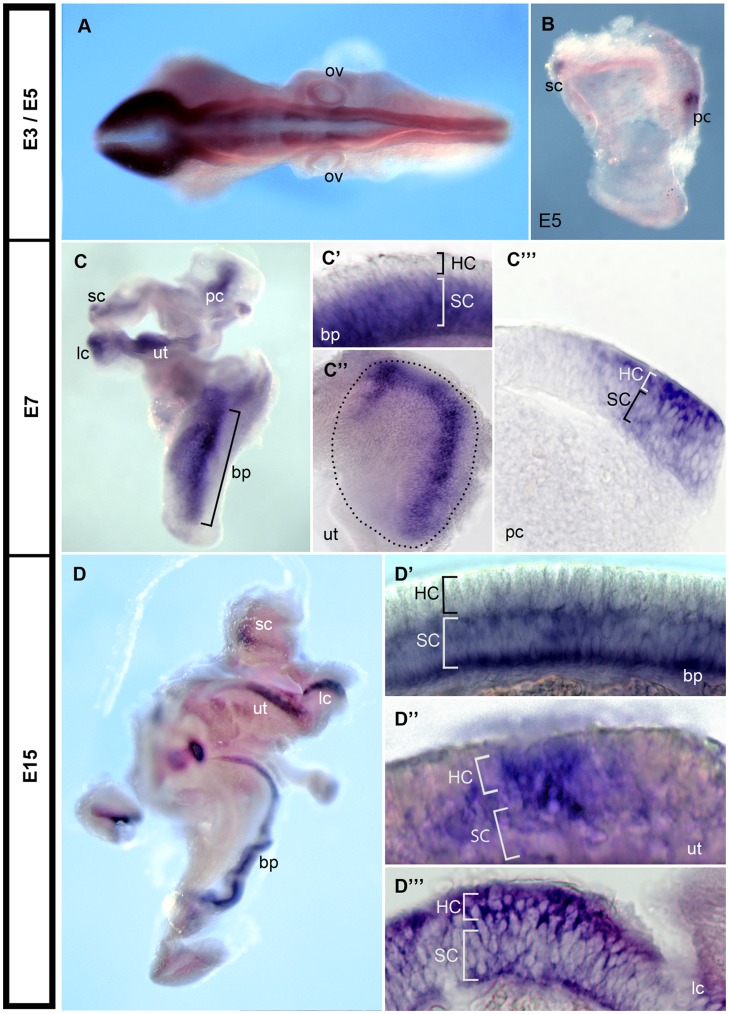
Expression of Sox21 during chicken inner ear development. (A) *Sox21* mRNA showed faint levels of expression at E3 in the otic vesicle. (B) Upregulation of *Sox21* expression was observed in the presumptive posterior crista and the presumptive superior crista regions at E5. (C) At E7 *Sox21* transcripts were detected in the basilar papilla, the utricle, the saccule and the posterior, superior and lateral cristae. (C’) in the utricle, *Sox21* is strongly expressed in the striola region (entire utricle is marked with black dotted line). (C’’) Transverse cryosection through the basilar papilla reveals *Sox21* transcripts are restricted to the support cell layer. (C’’’) Transverse cryosection through the utricle reveals that *Sox21* transcripts are enriched in the hair cell layer. (D) At E15 *Sox21* transcripts were still present in the basilar papilla, the utricle, the saccule and the posterior, superior and lateral cristae. D’) Transverse cryosection through the E15 basilar papilla reveals *Sox21* transcripts remain restricted to the support cell layer. (D’’) Transverse cryosection through the E15 utricle reveals *Sox21* transcripts are enriched in the hair cell layer. (D’’’) Transverse cryosection through the E15 cristae reveal *Sox21* transcripts are enriched in the hair cell layer. Picture shown is of a lateral crista; all cristae exhibited the same expression pattern. ov: otic vesicle; sc: superior crista; pc: posterior crista; lc: lateral crista; ut: utricle; bp: basilar papilla; HC: hair cell layer; SC: support cell layer.

We used *in situ* hybridization to investigate *Sox21* expression at different stages of chicken inner ear development. At embryonic day (E) 3, there was strong *Sox21* expression in the brain and neural tube, but only very faint expression in the otic vesicle (Fig. 1A). At E5, *Sox21* transcripts were detected in the inner ear, in the location of presumptive posterior and superior cristae (Fig. 1B). By E7, *Sox21* transcripts were clearly visible in all of the sensory epithelia (posterior, superior and lateral cristae, utricle, saccule) and in the auditory epithelium, the basilar papilla (Fig. 1C). In the basilar papilla, *Sox21* transcripts were restricted to the support cell layer and were absent from the hair cell layer (Fig. 1C’), which is consistent with Sox21 expression pattern in the mouse organ of Corti [24]. However in the striola region of the utricle (Fig. 1C’’), and in the cristae (Fig. 1C’’’), *Sox21* transcripts were preferentially located in the hair cell layer. This expression pattern was also found at E15 (Fig. 1D; 1D’; 1D’’; 1D’’’), when hair cell differentiation has stopped in the basilar papilla, but is still ongoing in vestibular patches. These results showed that *Sox21* is upregulated within sensory progenitors at the time of hair cell formation, however the cells with highest levels of *Sox21* expression are either hair cells in the vestibular patches, or support cells in the basilar papilla.

### Over-expression of Sox21 at Early Stages of Inner Ear Development Leads to a Loss of Prosensory Identity

**Figure 2:**
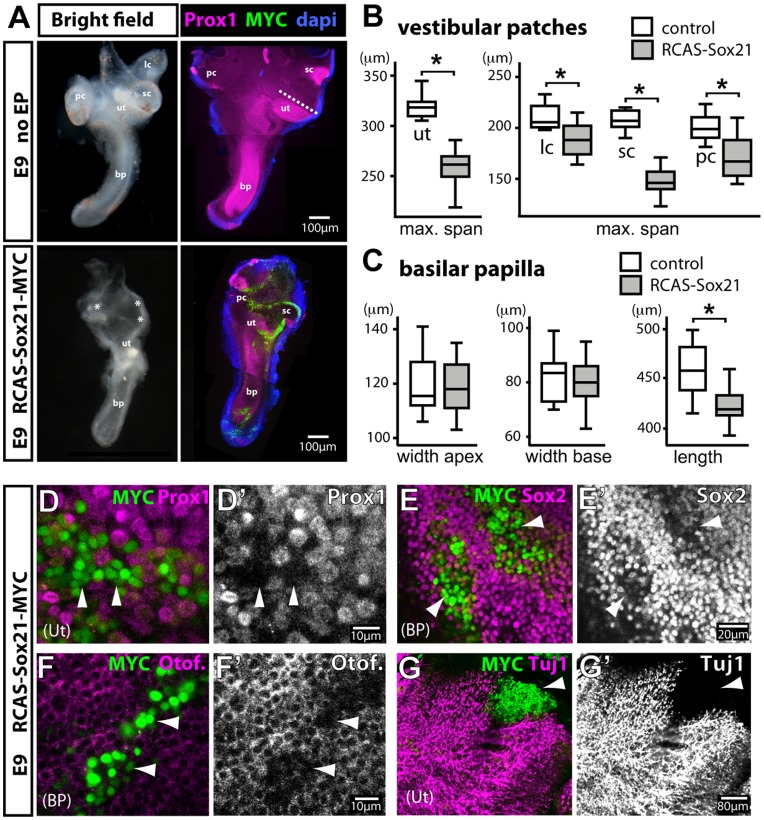
Over-expression of Sox21 at early stages of inner ear development leads to a loss of prosensory identity. (A) Brightfield and Prox1/Myc/Dapi immunostained views of inner ear dissected from E9 unelectroporated controls and RCAS-Sox21 electroporated embryos. Inner ears over-expressing Sox21 exhibit morphogenesis defects, including a reduction in the size of the cristae (white asterisks) and the presumptive utricle (marked as ”ut/sac” because identification is based on its position, and the reduced patch may also represent the saccule; images are composites of projections of distinct confocal stacks). Box plots of vestibular sensory patch maximum span (B; dashed line in panel A illustrates span of the utricle) and widths and length of the basilar papilla (C) in control (n = 12) and Sox21 (n = 13) transfected whole-mount preparations. Minimum, first quartile, median, third quartile and maximum are displayed. Prox1 (D–D’) and Sox2 (E–E’) expression is reduced in both vestibular and auditory sensory cells over-expressing Sox21 (white arrowheads). Immunostaining for otoferlin/HCA (F–F’) and Tuj1 (G–G’) show that cells over-expressing Sox21 from E2 onwards do not form hair cells or neurons and are not innervated. sc: superior crista; pc: posterior crista; lc: lateral crista; ut: utricle; bp: basilar papilla.

The expression data suggested that *Sox21* plays a role in sensory patch development and hair cell differentiation, and that this role may differ between the vestibular and auditory organs. To test the function of Sox21, we first used an RCAS retroviral vector to drive constitutive expression of a MYC-tagged version of chicken Sox21 protein in the developing inner ear. Following *in ovo* electroporation of the otic placode/cup with RCAS-Sox21-MYC plasmid DNA, the embryos were incubated until E9, a stage at which morphogenesis of the inner ear and hair cell production are well advanced. Sox21 over-expression produced a distinct and reproducible phenotype in which the vestibular part of the inner ear was systematically smaller than in control ears (n = 12/12). In the most extreme cases, all three cristae appeared smaller in size when compared with controls, and a single sensory patch of reduced size was in place of the utricle and saccule (n = 8/12). To quantify the differences in sensory patch size we measured in whole-mount preparations immunostained for Prox1 expression the maximum span of each vestibular patch, and the length and apical and basal width of the basilar papilla. We compared these measurements and found that all of the vestibular sensory patches were significantly reduced in size (t-test, p<0.05; control: n = 12, Sox21: n = 13; Fig. 2B). The length of the basilar papilla in Sox21-transfected samples was slightly shorter than that of controls (t-test, p<0.05; control: n = 12, Sox21: n = 13), however the widths of the basilar papilla, measured in basal and apical regions, were unchanged (Fig. 2C).

To assess the effects of Sox21 over-expression on sensory specification, we immunostained the samples for Prox1 and Sox2, which are markers of sensory progenitors and support cells in the inner ear [8], [9], [33], [34]. Prox1 expression was reduced or completely absent in Sox21 over-expressing cells when compared to neighbouring untransfected cells (Fig. 2 D–D’, white arrowheads). Likewise, Sox2 expression was consistently reduced in Sox21-MYC positive cells (Fig. 2 E–E’).

As they mature, hair cells downregulate Sox2 and Prox1 expression [9], [34]. Hence, the loss of Sox2 and Prox1 in cells infected with RCAS-Sox21 may have been an indication that these cells had differentiated into hair cells. To investigate this possibility we analysed the expression of two hair cell markers, the hair cell antigen (HCA) [29] and otoferlin [30], as well as beta-tubulin class III (Tuj1), which labels immature hair cells and the nerve fibres that innervate them [35]. In both the auditory and vestibular sensory patches, cells over-expressing Sox21 were not labelled with HCA/otoferlin antibodies (Fig. 2F–F’, white arrowheads). Nerve fibres, labelled by Tuj1, were absent from large patches of infection located within sensory epithelia, further confirming the absence of neurons or hair cells among Sox21-over-expressing cells (Fig. 2G–G’, white arrowhead). These results showed that over-expression of Sox21 in otic cells prevented them from adopting a prosensory fate.

### Temporal Control of Sox21 Over-expression Reveals a Potential Role in Hair Cell Formation in Vestibular Patches, but not in the Basilar Papilla

**Figure 3:**
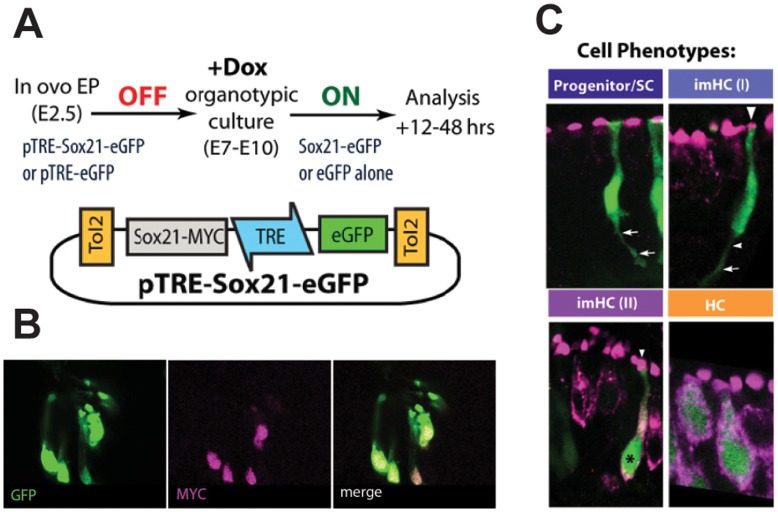
Tol2 system for Dox-inducible gene expression and classification of cell phenotypes. (A) The pTRE-Sox21-eEGFP Dox-inducible expression vector and experimental design for testing the effects of Sox21 induction at late stages of ear development. (B) MYC-tagged Sox21 was detected in the nuclei of all eGFP positive cells. (C) Classification of eGFP-positive cells into 4 phenotypes: uncommitted progenitor/support cell, in which otoferlin and HCA are not expressed, and with a cytoplasmic process contacting the basal lamina (arrows); immature hair cell type I (imHC (I)) in which HCA is present (arrowhead) but otoferlin is not, and which can exhibit a basal cytoplasmic process (arrows); immature hair cell type II (imHC (II)), with an elongated cell shape and both otoferlin and HCA expressed; Mature hair cell (HC), with a flask-shaped cell body and both otoferlin and HCA expressed.

Endogenous expression of Sox21 in the chicken inner ear does not reach high levels until E5 (Fig. 1), which coincides with the time of hair cell formation [36]. In our previous experiments, RCAS-mediated over-expression of Sox21 began at E2.5. This prevented us from assessing the specific effect of Sox21 over-expression upon hair cell versus support cell differentiation, at times when endogenous Sox21 is expressed in the inner ear. To overcome this problem, we used a Tet-on inducible Tol2 transposon system [37] in which a bidirectional tetracycline-responsive element (TRE) drives the expression of both eGFP and a gene of interest following doxycycline (Dox) treatment (Fig. 3A). Embryos co-electroporated at E2 with either pTRE-eGFP or pTRE-eGFP-Sox21 plasmids (along with plasmids encoding Tol2 transposase and the rtTA-M2 tet-on activator) were killed at E7–E10 and their inner ear maintained in vitro for up to 48 hours in the presence of Dox (see Fig. 3A for schematic). For ease of analysis we used the presence of eGFP as a marker for the presence of Sox21 in samples electroporated with pTRE-eGFP-Sox21. Immunostaining for MYC-tagged Sox21 confirmed that there was very good correlation with eGFP expression in Dox-treated samples (Fig. 3B). Analysis of cell morphology and molecular markers allowed us to assess whether the eGFP-positive cells were hair cells or progenitor/support cells. The eGFP-positive cells were counted and categorised into 4 phenotypic classes according to their morphologies and expression of HCA and otoferlin (Fig. 3C, see legend for description of phenotypic classes). Because the subcellular localisation of HCA and otoferlin differs within hair cells, and was not modified by Sox21 overexpression induced by Dox treatment (data not shown), we labelled the two epitopes with the same secondary antibody in these experiments.

**Figure 4:**
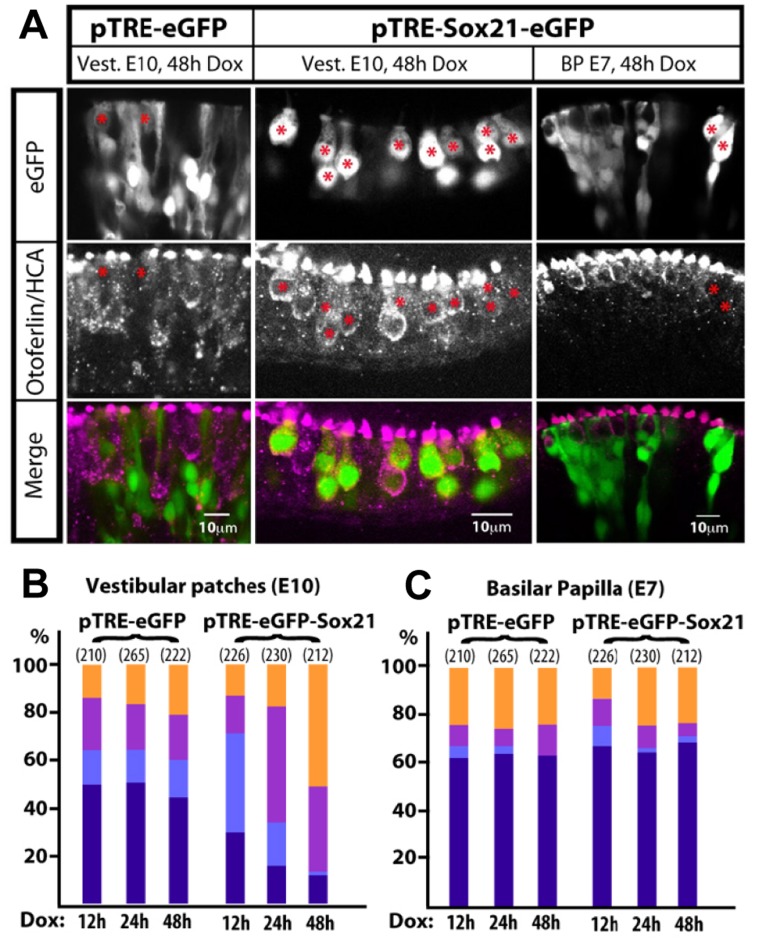
Induction of Sox21 expression at late embryonic stages promotes hair cell formation in vestibular patches, but not in the basilar papilla. (A) cultures of transfected inner ear tissue treated for 48 hours with Dox, then immunostained for otoferlin/HCA. With the pTRE-eGFP vector, eGFP-positive cells are mainly uncommitted progenitor/support cell types but include some hair cells (red asterisks). Induction of Sox21-eGFP in vestibular sensory epithelia for 48 hours results in a large majority of induced cells exhibiting a hair cell phenotype (red asterisks). In the basilar papilla, induction of Sox21-eGFP for 48 hours produces a phenotype indistinguishable from that of the control. (B–C) Phenotypic distribution (colors correspond to those used in Figure 3D) of eGFP-positive cells after 12, 24 and 48 hours Dox treatment. In vestibular sensory epithelia, Sox21 induction caused a strong shift towards hair cell phenotypes over time. Total cell counts from 3 separate ear samples are shown above bars.

Induction of Sox21 over-expression was performed at E10 in vestibular (utricle and crista) epithelia. In these samples, we found the majority of pTRE-eGFP transfected cells had support cell rather than hair cell morphologies. In contrast, the majority of cells transfected with pTRE-eGFP-Sox21 had hair cell morphology and were otoferlin and HCA-positive (Fig. 4A). Surprisingly, this result was not observed in organotypic cultures of the auditory epithelium, the basilar papilla. In this instance the induction of Sox21 over-expression was performed at E7, at a time when mitotic sensory progenitors are still present and few auditory hair cells have differentiated [29]. After 48 hours, the phenotypic distribution of Sox21-induced cells appeared very similar to that of samples transfected with the pTRE-eGFP construct (Fig. 4A).

To investigate the timing of Sox21 effects on hair cell differentiation, we analysed the molecular and morphological phenotype of eGFP-positive cells within the vestibular and auditory sensory patches after 12, 24 and 48 hours in Dox. Three independent samples were analysed for each time point. Cell counts were then pooled before the percentages of the four cell phenotypes were calculated. In agreement with previous work [30], we noted that expression of HCA at the apical surface always preceded that of otoferlin within the cytoplasm of immature hair cells (Fig. 3C). In controls transfected with pTRE-eGFP, a comparable distribution of cell phenotypes was observed across specimens at the three time points (Fig. 4B,C). In vestibular epithelia, the most common eGFP-induced cell type was the uncommitted progenitor/support cell type, with lower percentages of hair cell progenitors, immature hair cells and mature hair cells observed. In contrast, the proportion of Sox21-induced supporting/progenitor cells decreased as the time spent in the presence of Dox increased (Fig. 4B). After 12 hours of Dox treatment, the majority of Sox21-induced cells in the vestibular samples were very immature hair cells (44.7%) and by 48 hours of treatment, the majority were either immature or mature hair cells (83.1%; Fig. 4B). These data suggested that, at least in the vestibular epithelia, induction of Sox21 was able to bias progenitor cells towards a hair cell fate.

The induction of Sox21 in cultures of E7 basilar papilla did not have such effect. The pTRE-eGFP and pTRE-eGFP-Sox21 transfected cells exhibited similar distribution of cell phenotypes after 12, 24 or 48 hours of Dox treatment. In all samples, the most common phenotype was that of supporting/progenitor cells (63–68%) and the percentage of mature hair cells remained comparable between the control and experimental samples after 48 hours of Dox treatment (∼23%; Fig. 4C).

### Induction of Sox21 Causes a Reduction in Sox2 Expression

**Figure 5:**
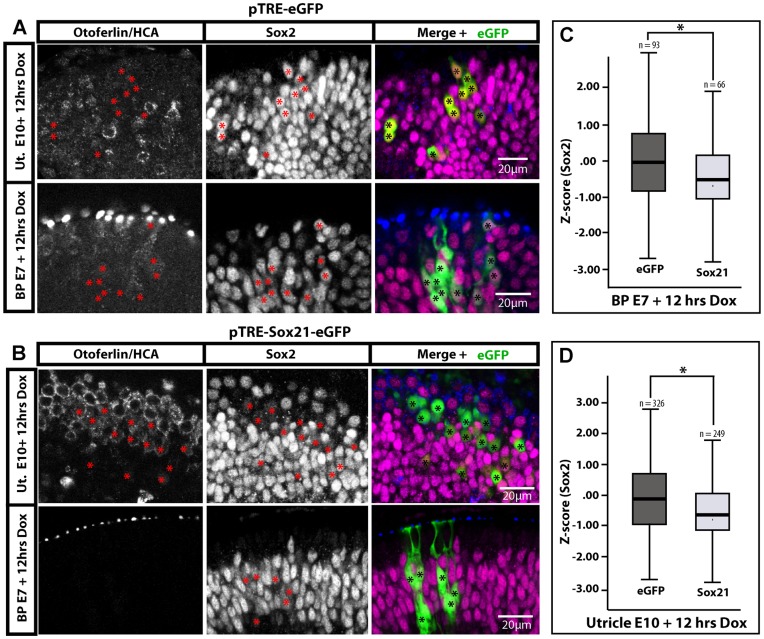
Short induction of Sox21 reduces Sox2 expression in organotypic cultures of E10 utricle and E7 basilar papilla. (B) Representative images of Sox2 immunostaining in pTRE-Sox21-eGFP samples after 12 hours of Dox treatment. Induced cells are marked with asterisks, and tend to exhibit reduced levels of Sox2 expression compared to neighbouring untransfected cells (C). Box plot of Z-score values [Z = (x-mean)/stdev] for levels of Sox2 expression in supporting/progenitor cells of the basilar papilla (B; 3 samples) or of the utricle (C; 5 samples), expressing either eGFP only or Sox21-eGFP. Outliers, minimum, first quartile, median, third quartile and maximum are displayed, n = numbers of transfected cells. Sox2 expression levels were significantly lower in Sox21-eGFP expressing cells than in eGFP expressing cells in both the auditory (Mann-Whitney U = 14873; p = 0.00) and vestibular (Mann-Whitney U = 32335; *p* = 0.00) epithelia.

Sox2 can antagonize hair cell differentiation [9], [12] and our previous results showed that Sox21 could inhibit Sox2 expression at early stages of inner ear development. Hence, it seemed possible that transient over-expression of Sox21 could result in a down-regulation of Sox2 expression, which in turn might bias uncommitted progenitor cells towards a hair cell fate. To test this hypothesis, we analysed Sox2 expression in vestibular and auditory organs transfected with either pTRE-eGFP or pTRE-eGFP-Sox21 and treated for 12 hours with Dox (Fig. 5). Despite important cell-to-cell variations, there was no apparent difference in the levels of Sox2 expression between eGFP-positive and eGFP-negative cells in pTRE-eGFP transfected samples (Fig. 5A). On the other hand, levels of Sox2 expression appeared frequently reduced in both hair cells and uncommitted progenitor/support cells over-expressing eGFP-Sox21 when compared to neighbouring untransfected cell types (Fig. 5B). To ascertain this effect, we measured the levels of Sox2 expression within individual progenitor/support cells with a basal nucleus in samples transfected with either Sox21-eGFP or eGFP alone (Fig. 5B,C; see methods). The normalized intensity values for Sox2 expression were lower and statistically different in Sox21-eGFP-expressing cells when compared to eGFP-expressing cells in both auditory (Sox21-eGFP/n = 66; eGFP/n = 93; Mann-Whitney U = 2268; *p = *0.005) and vestibular (Sox21-eGFP/n = 249; eGFP/n = 326; Mann-Whitney U = 32335; *p* = 0.000) epithelia. This suggested that elevating the levels of Sox21 expression could inhibit Sox2 expression in sensory progenitors and support cells.

### Induction of Sox21 Reduces Endogenous Levels of Notch Activity

**Figure 6:**
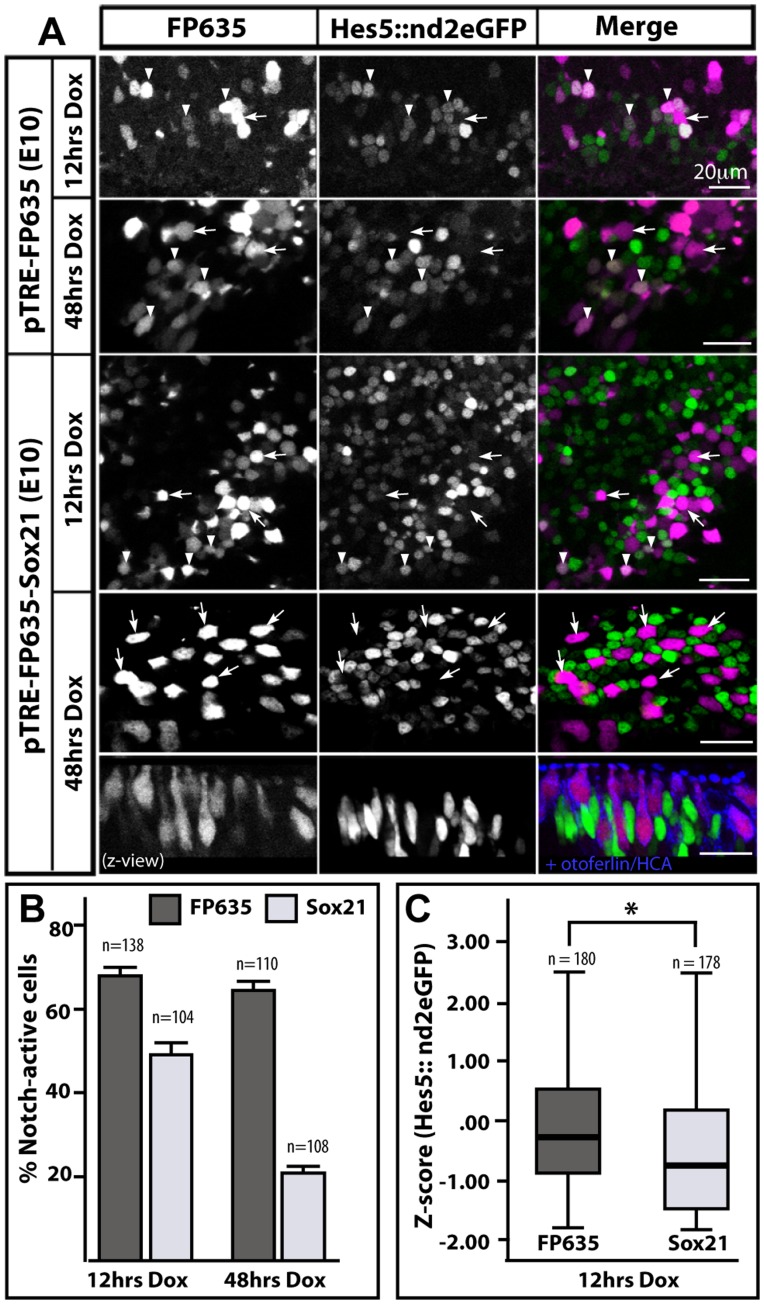
Induction of sox21 causes a progressive reduction in levels of Notch activity. (A) surface views of E10 vestibular patches transfected with the pT2K-Hes5::nd2eGFP reporter and either pTRE-FP635 (control) or pTRE-FP635-Sox21 and treated in vitro with Dox for 12 or 48 hours. In control samples, the proportion of FP635-positive cells that were either positive (arrowheads) or negative (arrow) for nuclear Hes5::nd2eGFP expression was comparable after 12 or 48 hours Dox treatment. In contrast, the number of Sox21-FP635 induced cells positive for Hes5::nd2eGFP expression was reduced at both 12 hours and 48 hours of Dox treatment. In the 48 hours example shown, all FP635-Sox21 are negative for Notch activity (white arrows); the transverse reconstruction of the same region (z-view panel) also demonstrate expression of otoferlin/HCA in FP635-Sox21 induced cells. (B) Graph showing the proportion of Notch-active (mean intensity of nuclear d2eGFP signal >500 above background in a 12-bits image) cells among cells induced for FP635-only (FP635) or FP635-Sox21 (Sox21) expression after 12 and 48 hours Dox treatment; n = total number of transfected cells analysed in 3 samples. Standard error bars are shown. (C) Box plots of Z-scores values for Hes5::nd2eGFP fluorescence levels in supporting/progenitor cells transfected with either pTRE-FP635 (n = 180) or pTRE-Sox21-FP635 (n = 178) and treated for 12 hours with Dox. Minimum, first quartile, median, third quartile, and maximum are displayed. There were significantly lower levels of Hes5::nd2eGFP fluorescence in Sox21-FP635 expressing cells than in FP635 expressing cells (Mann-Whitney U = 11232; *p* = .00; n = total number of transfected cells analysed in 3 samples).

**Figure 7:**
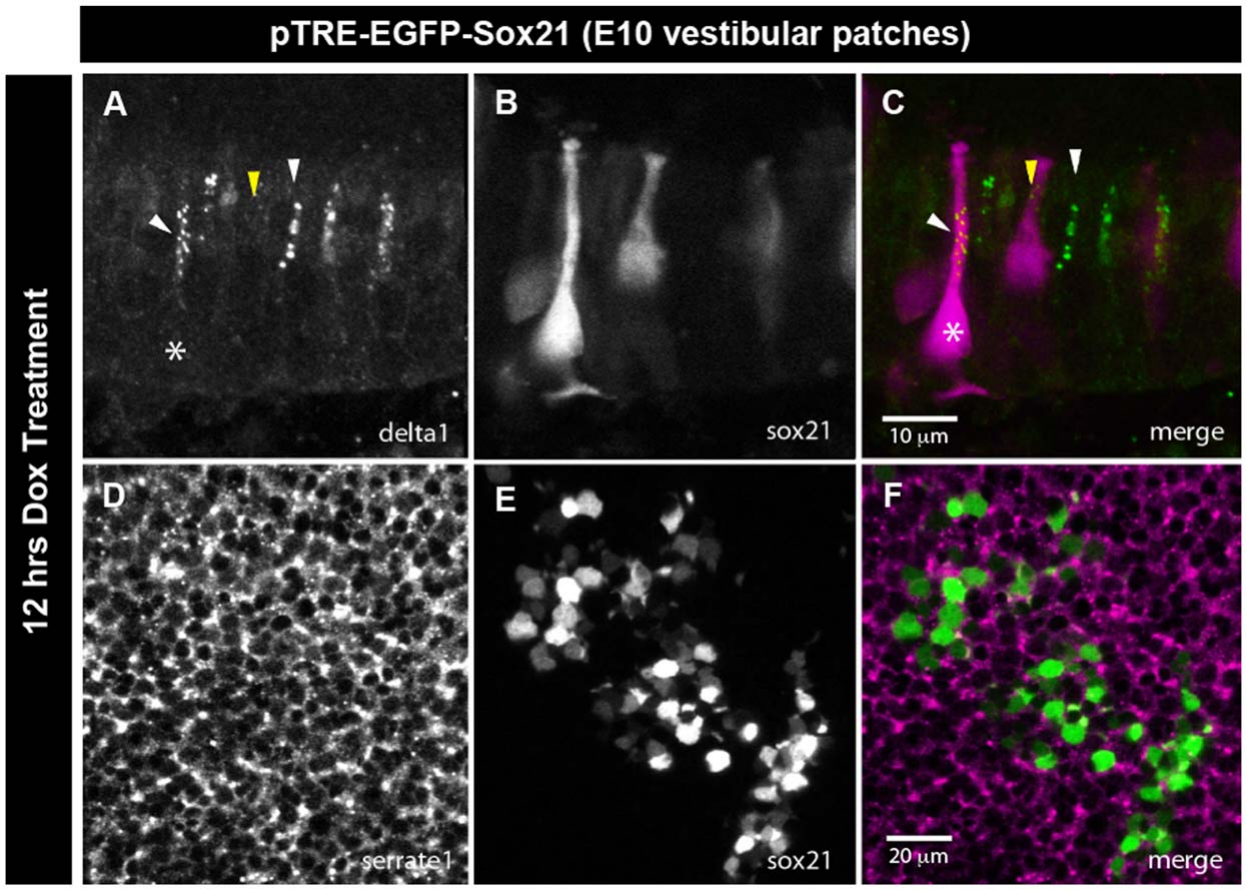
Overexpression of Sox21 does not influence Delta1 and Serrate1 expression. (A–C) After 12 hours Dox treatment, Delta1 expression does not differ between immature hair cells overexpressing Sox21, and control untransfected immature hair cells (white arrowheads denote the location of immature hair cells; white asterisk denotes Sox21 induced cell. Mature hair cells overexpressing Sox21 do not continue to exhibit Delta1 expression (yellow arrowhead), consistent with untransfected mature hair cells. (D–F): Apical view of Serrate1 expression shows no obvious changes in expression levels between regions of induced Sox21 overexpression and untransfected regions.

Hair cell fate decisions are regulated by lateral inhibition: the precursor cells that down-regulate Notch activity become hair cells and express Delta1, and signal to neighbouring cells to remain as mitotic progenitors or to differentiate into support cells by activating their Notch receptors [5], [6]. To determine the potential effects of Sox21 on Notch signalling, we used a Tol2 pT2K-Hes5::nd2EGFP reporter construct, in which the mouse Hes5 promoter [27] regulates the expression of a short-lived, nuclear-localized form of eGFP. This Hes5 reporter is sensitive to Notch signalling throughout the developing chicken inner ear, and can be used to monitor reductions in endogenous levels of Notch activity (E. Chrysostomou, J. Gale and N. Daudet, in press). We focused our analysis on vestibular patches in which induction of Sox21 had clear effects on hair cell differentiation. We co-electroporated either the pTRE-FP635 or pTRE-FP635-Sox21 (these inducible constructs included the red fluorescent protein Turbo-FP635 instead of eGFP to identify induced cells) with the pT2K-Hes5::nd2eGFP reporter at E2, let the embryos develop to E10 stages, then dissected and cultured vestibular patches from three independent samples in the presence of Dox. After 12 hours of treatment, we found endogenous Notch activity (defined as Hes5::nd2eEGFP levels >500 above background in a 12-bits image) in 68.1% of control FP635-induced cells (n = 94/138), versus only 49% of FP635-Sox21 induced cells (n = 51/104; Fig. 6A,B). This difference was even greater after 48 hours: Notch activity was observed in an average of 64.5% of FP635 induced cells (n = 71/110), but in only 21.2% of FP635-Sox21 cells (n = 23/108; Fig. 6B). In some cases, a striking checkerboard-like pattern of FP635-Sox21 induced cells surrounded by strongly nd2EGFP-positive cells was observed (Fig. 6A, lower panels). We next quantified Hes5::nd2EGFP fluorescence levels of individual progenitor/support cells after 12 hours of Dox treatment. In control samples, there was no statistically significant difference in levels of Hes5::nd2EGFP fluorescence of FP635-induced versus non-induced cells (Mann Whitney U test; p = 0.000; total n nuclei = 180 transfected/432 untransfected in n = 3 samples). However, there was a significant decrease (Mann-Whitney U test = 11232; p = 0.000) in the levels of nd2EGFP expression in FP635-Sox21 transfected cells (n = 178; n = 3 samples) when compared to FP635-transfected cells (n = 180; n = 3 samples) (Fig. 6C). On the other hand, expression of the Notch ligands Delta1 and Serrate1 did not appear to differ between control and Sox21-induced cells at this time point (Fig. 7A–F), suggesting that the reduction in endogenous Notch activity was not consecutive to stronger lateral inhibition delivered by Sox21-induced cells. These data suggested that artificial elevation of Sox21 expression in vestibular sensory progenitors results in a rapid reduction of endogenous Notch activity, which is consistent with the progression of Sox21-induced cells towards a hair cell phenotype.

### Induction of Sox21 does not Upregulate Activity of the Atoh1::nTomato Reporter

**Figure 8:**
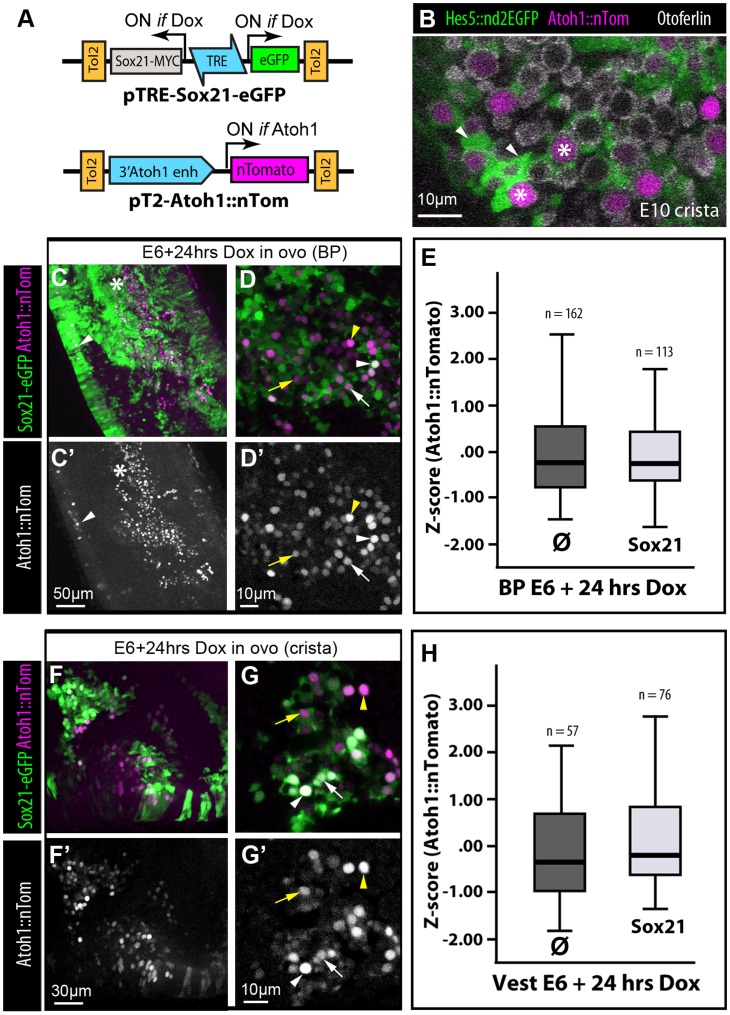
Fluorescence of the pT2K-Atoh1::nTomato reporter is not elevated in Sox21-induced cells. (A**)** Schematic representation of the Tol2 Dox-inducible Sox21 and the Tol2 Atoh1 reporter constructs. (B**)** A vestibular crista transfected with pT2K-Hes5::d2eGFP and the pT2K-Atoh1::nTomato reporters and immunostained with otoferlin antibodies; the Atoh1::nTomato fluorescence is strong in hair cells (asterisks), but not in Hes5::d2eGFP positive cells surrounding them (arrowheads). (C–D**)** Surface views of a BP transfected with pTRE-Sox21-eGFP and pT2K-Atoh1::nTomato at E2 and treated *in ovo* for 24 hrs with Dox at E6. Atoh1::nTomato-positive cells were found primarily in the central-distal region of the BP (asterisk), but a few Sox21-induced cells in the lateral wall were also positive (arrowheads in C–C’). At higher magnification (D–D’), note that the levels of Atoh1::nTomato fluorescence varied greatly in both Sox21-induced (white arrows and arrowheads) and non-induced (yellow arrows and arrowheads) cells. (E) Box plots of Z-scores values for Atoh1::nTomato fluorescence levels in untransfected (ø) versus Sox21-induced cells in the basilar papilla. Minimum, first quartile, median, third quartile, and maximum are displayed. There was no significant difference between the two categories of cells (Mann-Whitney U = 9818; *p* = 0.305; n = number of cells analysed in 2 samples). (F–G**)** surface views of a crista transfected with pTRE-Sox21-eGFP and pT2-Atoh1::nTomato at E2 and treated *in ovo* for 24 hrs with Dox at E6. Variations in levels of Atoh1 reporter fluorescence are also clearly visible. (H**)** Box plots of Z-scores values for Atoh1::nTomato fluorescence levels in untransfected (ø) versus Sox21-induced cells in vestibular epithelia. Minimum, first quartile, median, third quartile, and maximum are displayed. There was no significant difference between the two categories of cells (Mann-Whitney U = 2560; *p* = 0.073; n = number of cells analysed in 2 samples).

The Atoh1 transcription factor is required for hair cell formation [38], and artificial induction of Atoh1 expression can promote hair cell formation in the immature inner ear [39]. To test whether Sox21 might regulate Atoh1 gene expression, we investigated the consequences of a relatively short induction of Sox21 expression on the activity of a fluorescent reporter of Atoh1 expression, pT2K-Atoh1::nTomato (Fig. 8A). This Tol2 construct consisted of the 3′ enhancer of the mouse Atoh1 gene [40], regulating the expression of a nuclear-localized Tomato fluorescent protein. Previous experiments showed that high levels of nTomato are present in the nuclei of pT2K-Atoh1::nTomato transfected hair cells, identifiable by HCA and otoferlin expression, from E6 onwards (data not shown and Fig. 7B).

Following electroporation of the Atoh1 reporter and the pTRE-Sox21-eGFP constructs at E2, embryos were treated *in ovo* with Dox at E6 to induce Sox21, and then incubated for a further 15 (n = 4) and 24 (n = 4) hours. At both time points and in both vestibular and auditory epithelia, we found that levels of Atoh1::nTomato fluorescence were variable from cell to cell, but did not appear elevated in Sox21-eGFP expressing cells (Fig. 8C–H). In both vestibular and auditory regions, Atoh1::nTomato positive cells were located within the sensory epithelia, with the exception of a subset of Sox21-eGFP expressing cells in the lateral wall of the basilar papilla (n = 3/4 samples; arrowhead in Fig. 8C–C’). At 24 hours post-induction, the distribution of the mean values of Atoh1::nTomato fluorescence of Sox21-eGFP expressing and non-expressing cells (Fig. 8E, 8H) were not statistically different in the basilar papilla (total n nuclei = 113 transfected/162 untransfected; Mann-Whitney U test = 9818, p = 0.305) and in the vestibular epithelia (total n nuclei = 76 transfected/57 untransfected; Mann-Whitney U test = 2560, p = 0.073). These results suggested that Sox21 does not directly regulate Atoh1 gene expression.

## Discussion

The SoxB1 transcription factor Sox2 has essential roles during sensory development in the inner ear, but the mechanisms regulating its expression or function are still poorly understood. Here, we found that the SoxB2 family member Sox21 could be an important regulator of Sox2 function in the inner ear. We show that over-expression of Sox21 leads to inhibition of Sox2 expression and Notch activity, two of the major players implicated in both prosensory specification and the terminal differentiation of hair cells and support cells. Furthermore, over-expression of Sox21 has the ability to promote hair cell formation in a context-dependent manner. Thus, we suggest that antagonistic interactions among SoxB family transcription factors could regulate sensory cell development in the inner ear.

### Context and Dosage Determine the Function of SoxB Transcription Factors in the Inner Ear

A strict regulation of the level of Sox2 expression is critical for its functions. In mammals, the complete absence of the *Sox2* gene is embryonic lethal [41], and the phenotypes resulting from partial inactivation of *Sox2* expression are highly sensitive to dosage in the central nervous system [42]. Similarly, in the inner ear, complete absence of *Sox2* expression in Lcc/Lcc mutant mice leads to a total failure of sensory development, but partial formation of sensory patches occurs in the Ysb/Ysb mutant that has reduced levels of *Sox2* [10]. Furthermore, hair cell numbers are increased in the organ of Corti of *Sox2* hypomorphic mice, suggesting that partial reduction of *Sox2* expression can enhance hair cell formation [12]. Here we found that a related member of the SoxB family, the SoxB2 subgroup member Sox21, is a potent regulator of sensory cell formation in the inner ear.

Over-expression of Sox21 from an early stage of ear development severely disrupted inner ear morphogenesis. Otic cells over-expressing Sox21 failed to express Sox2 and the prosensory marker Prox1, and the drastic morphological abnormalities of sensory and non-sensory structures of the vestibular region were reminiscent of those seen in the *Sox2* mutant mice [10]. This suggests that Sox21 can antagonize the early phase of Sox2 activity linked to prosensory specification in the inner ear. Nevertheless, our expression data indicate that Sox21 acts at later stages of ear development, when hair cells are produced. Using a Dox-inducible system to specifically activate Sox21 expression at late embryonic stages, we found that Sox21 can promote hair cell differentiation in embryonic vestibular epithelia, but not in the basilar papilla. On the other hand, over-expression of Sox21 reduced Sox2 expression in both the vestibular epithelia and the basilar papilla. This suggests that SoxB transcription factors exert a greater influence on hair cell fate decisions in the vestibular sensory epithelia than in the auditory sensory epithelium. The variation in the expression of endogenous *Sox21* between vestibular and auditory epithelia further suggests a difference in the role of SoxB transcription factors in the two sensory systems. In the vestibular epithelia, *Sox21* is enriched in the hair cell layer, which is consistent with a “pro hair cell” role for the endogenous Sox21. In the auditory epithelia of the chicken and the mouse [24], *Sox21* is progressively restricted to the support cell layer, which suggests an alternative function, possibly in fine-tuning the function or expression levels of Sox2 within support cells. This could explain why the organ of Corti of *Sox21* knock-out mice has only occasional and subtle defects in hair cell patterning [24]. However, reduced or delayed hair cell formation may occur in the vestibular epithelia of the Sox21 mutant mice, which have not been examined yet.

Finally, it is worth noting that the antagonistic effect of Sox2 on hair cell differentiation is not systematic either: in the adult vestibular system of birds and mammals, at least one subtype of hair cell appears to maintain high levels of Sox2 expression [7], [43]. In the zebrafish inner ear, Sox2 is not required for hair cell formation and it does not prevent the overproduction of hair cells induced by Atoh1 over-expression [44], [45]. Hence, the functions of SoxB transcription factors and the outcomes of their interactions in the inner ear are strongly influenced by dosage and the cellular context in which they operate.

### Sox21 could Promote Vestibular Hair Cell Formation by Regulating Sox2 Expression and Notch Activity

The Sox2 and Sox21 proteins have similar DNA binding domains but exhibit opposite transcriptional activities, suggesting that their antagonistic functions during neurogenesis could stem from their contrasting effects on a common set of target genes [22], [23]. In the inner ear, these targets could include some genes required for hair cell versus support cell differentiation, such as *Atoh1* [38], [46], [47]. However our experiments using a reporter of Atoh1 expression suggest that Sox21 does not strongly regulate Atoh1 gene expression. On the other hand, we found that sustained as well as transient induction of Sox21 can inhibit Sox2 expression, a result similar to that previously obtained in human glioma cell lines [48]. Given that Sox2 is thought to antagonize Atoh1 function in the inner ear [12], the reduction of Sox2 levels induced by Sox21 could indirectly promote hair cell differentiation. Artificial induction of Sox21 also resulted in a progressive loss of Hes5::nd2EGFP fluorescence in transfected cells, which was already visible 12 hours after the onset of Dox treatment. This cell-autonomous reduction in Notch activity could either reflect the rapid progression of Sox21-induced cells towards a hair cell fate, or be partly responsible for this process because of the well-known implication of Notch signalling in the lateral inhibition of hair cell formation [5]. How could Sox21 inhibit Notch activity? One interesting possibility, suggested by Notch1 promoter studies and ChiP experiments in the mouse CNS, is that Sox2– and by extension, interactions among SoxB family members- could regulate the expression of the Notch1 receptor [42]. Future studies will be needed to determine whether this is the case in the inner ear. It will be equally important to identify the upstream factors directing Sox21 expression in sensory progenitor cells, particularly since analysis of the Sox21 promoter region has revealed highly conserved elements that direct its expression to the zebrafish CNS and inner ear [49].

### SoxB Transcription Factors and Hair Cell Regeneration

Our findings establish Sox21 as an important regulator of hair cell differentiation. This in turn makes Sox21 a potential target for hair cell regeneration therapies. Fish and birds can regenerate inner ear hair cells throughout life, while in mammals limited capacities for post-traumatic regeneration exist in the vestibular organs, but not in the mature organ of Corti [50]–[52]. Over-expression of the “pro-hair cell” transcription factor Atoh1 [38] is currently the favoured approach for hair cell regeneration therapies. Atoh1 can induce the formation of supernumerary hair cells in the embryonic inner ear [39], [53]–[55], and it promotes hair cell production in mature vestibular epithelia [56]. However, the competence of Atoh1-transfected embryonic progenitors to differentiate into hair cells is regionally restricted and regulated by additional factors, including Sox2 [45], [47]. As they mature, support cells of the organ of Corti gradually lose their potential to convert into hair cells even upon forced expression of Atoh1 [57], [58] or to proliferate [59] arguing that Atoh1 gene therapy alone will not be sufficient to induce complete regeneration in this tissue [51]. In vestibular epithelia, the competence of support cells to regenerate new hair cells may be limited by a number of inhibitory signals, such as Notch activity [60], [61] and perhaps Sox2. If this was the case, the combination of Atoh1 gene therapy with additional factors antagonizing Notch and SoxB1 activities, such as Sox21, could improve hair cell regeneration processes in the mammalian inner ear.
